# Enrichment of ultraconserved elements among genomic imbalances causing mental delay and congenital anomalies

**DOI:** 10.1186/1755-8794-3-54

**Published:** 2010-11-23

**Authors:** Francisco Martínez, Sandra Monfort, Mónica Roselló, Silvestre Oltra, David Blesa, Ramiro Quiroga, Sonia Mayo, Carmen Orellana

**Affiliations:** 1 Unidad de Genética y Diagnostico Prenatal; Hospital Universitario La Fe; Valencia, 46009; Spain; 2 Servicio de Análisis de Microarrays, Centro de Investigación Príncipe Felipe (CIPF), Valencia, Spain

## Abstract

**Background:**

The ultraconserved elements (UCEs) are defined as stretches of at least 200 base pairs of human DNA that match identically with corresponding regions in the mouse and rat genomes, albeit their real significance remains an intriguing issue. These elements are most often located either overlapping exons in genes involved in RNA processing or in introns or nearby genes involved in the regulation of transcription and development. Interestingly, human UCEs have been reported to be strongly depleted among segmental duplications and benign copy number variants (CNVs). However no comprehensive survey of a putative enrichment of these elements among pathogenic dose variants has yet been reported.

**Results:**

A survey for UCEs was performed among the 26 cryptic genomic rearrangements detected in our series of 200 patients with idiopathic neurodevelopmental disorders associated to congenital anomalies. A total of 29 elements, out of the 481 described UCEs, were contained in 13 of the 26 pathogenic gains or losses detected in our series, what represents a highly significant enrichment of ultraconserved elements. In addition, here we show that these elements are preferentially found in pathogenic deletions (enrichment ratio 3.6 vs. 0.5 in duplications), and that this association is not related with a higher content of genes. In contrast, pathogenic CNVs lacking UCEs showed almost a threefold higher content in genes.

**Conclusions:**

We propose that these elements may be interpreted as hallmarks for dose-sensitive genes, particularly for those genes whose gain or loss may be directly implied in neurodevelopmental disorders. Therefore, their presence in genomic imbalances of unknown effect might be suggestive of a clinically relevant condition.

## Background

A current challenge of medical genetics is aimed to disentangle the relationship between the genomic data provided by the introduction of array comparative genomic hybridization (array-CGH) and the phenotypic consequences of the gains and losses observed along the whole genome. The classical criteria of assigning a pathogenic condition to any *de novo *alteration cannot be always applied, as exceptions in both senses can occur: benign copy number variants (CNVs) can arise *de novo *(unpublished results) while some pathogenic alterations are associated to such a broad phenotypic spectrum that they may have been inherited from apparently healthy parents [1]. Comparative genomics may provide an invaluable tool in the task of differentiating benign from pathogenic CNVs or, in other words, to evaluate which genes may be dose-sensitive or not. In this context, the real significance of the ultraconserved elements (UCEs) in the human genome remains an intriguing issue. There are 481 UCEs, defined as stretches of at least 200 base pairs of human DNA that match identically with corresponding regions in the mouse and rat genomes [2]. They are widely distributed in the genome (on all the chromosomes except chromosomes 21 and Y) and are often found in clusters. For unknown reasons, these regions are under a negative selection much stronger than that operating in coding sequences and have been evolutionarily conserved for 300 million years, before mammal and bird ancestors diverged [3]. Of the 481 ultraconserved elements, 111 overlap the mRNA of a known gene, 256 show no evidence of transcription, and for the remaining 114 the evidence for transcription is inconclusive. These elements are most often located either overlapping exons in genes involved in RNA processing or in introns or nearby genes involved in the regulation of transcription and development. These elements are frequently found in genes post-transcriptionally regulated by alternative splicing events of exons with premature stop codons. Accordingly, the extreme genomic conservation has been associated to regulatory splicing events maintaining tightly regulated levels of RNA-binding proteins [4]. On the other hand, intergenic elements are frequently flanked by developmental genes, in particular for genes involved in early developmental tasks, suggesting that many of the associated ultraconserved elements may be distal enhancers of these early developmental genes [2].

Functional studies suggested that these elements show a tissue-specific in vivo enhancer activity in a mouse transgenic reporter assay that tended to recapitulate aspects of the expression pattern found in genes that were in their proximity [5]. On the other hand, the removal of four of these UCEs, located near genes that exhibit marked phenotypes in murine models, failed to reveal any overt variation of growth, longevity, pathology or metabolism. As the authors concluded, these results indicate that extreme sequence constraint does not necessarily reflect crucial functions required for viability, although not all the possible phenotypic impact was evaluated [6].

Interestingly, human UCEs have been reported to be strongly depleted among segmental duplications and benign copy number variants [7]. However, no comprehensive survey of a putative enrichment of these elements among pathogenic dose variants, and more specifically among those rearrangements directly related with neurodevelopmental disorders, has yet been attempted.

## Results

We performed an inspection for the 481 previously reported ultraconserved elements among the cryptic genomic rearrangements detected in our series of patients with idiopathic neurodevelopmental disorders associated to congenital anomalies. We found a total of 29 different elements contained in 13 out of the 26 pathogenic gains or losses detected in our series (Table 1). These dose alterations span variable sizes, ranging from 210 Kb to 13.4 Mb, which altogether represent almost 83 Mb of non-overlapping genomic sequence, or about 2.67% of the human genome. Assuming a random distribution for the UCEs along the genome, about 12.8 (481 × 0.0267) elements of this kind would be expected to be found. The difference between the number of observed and expected elements represents a highly significant enrichment of these elements among the pathogenic CNVs (chi-square = 20.32; P < 0.00001). The proportion between exonic and non-exonic UCEs in our series (8 vs. 17) is quite similar to the global proportion (111 vs. 256), what suggests that neither type is over-represented in pathogenic imbalances.

**Table 1:** Pathogenic imbalances and UCEs

Case	Imbalance	**Chr**.	Region	Initial pos.^a ^(Mb)	Final pos.^a ^(Mb)	Size (Kb)	Inheritance	Origin	Ultraconserved elements^b^
3	Loss	1	q24-25	167.024	172.151	5,000	De novo	Maternal	
15	Loss	1	q44	242.638	243.791	1,153	De novo	Paternal	uc.45-uc.46
88	Gain	2	p22.2-23.3	24.374	37.745	13,371	De novo	Paternal	uc.49
C2	Loss	2	q31	177.246	180.516	3,270	De novo	Paternal	uc.109
92	Loss	3	q13.2-13.3	113.681	116.922	3,242	De novo	Maternal	uc.119-uc.122
C1	Loss	3	q29	197.293	198.467	1,174	Inherited	Maternal	
18	Loss	5	q14.3	86.142	90.112	3,970	De novo	Paternal	uc.163-uc.167
2	Loss	6	q16	93.933	103.287	9,200	De novo	Paternal	uc.194, uc.195-uc.200, uc.201, uc.202
7	Loss	6	q16	93.930	104.244	10,314	De novo	Paternal	uc.194, uc.195-uc.200, uc.201, uc.202
M2	Loss	7	p21.2	~19.120	~19.700	~580	De novo	n.d.	
C4	Gain	7	q11.3	73.481	74.981	1,500	De novo	n.d.	
C3	Gain	8	q12	59.536	62.526	2,990	De novo	Paternal	uc.239
78	Loss	11	q13.5	75.730	83.639	7,909	De novo	Paternal	uc.331
16	Loss	13	q32	93.423	98.967	5,544	De novo	Paternal	uc.355, uc.356
C5	Gain	14	q11.2	18.832	23.660	4,828	De novo	Maternal	
9	Loss	15	q23	67.908	69.198	1,290	Inherited	Maternal	uc.392
M1	Loss	17	p11.2	~18.400	~20.200	~1,800	De novo	n.d.	
M3	Loss	17	q21.31	~41.100	~41.600	~500	De novo	n.d.	
63	Gain	19	p13.3	1.904	6.860	4,956	De novo	Maternal	
10	Loss	20	q12-13.11	39.463	42.008	2,540	Inherited	Paternal	uc.456
71	Gain	22	q11	17.276	19.230	1,954	Inherited	Paternal	uc.457
41	Gain	X	p11.23	45.063	48.922	3,859	De novo	Paternal	
42	Gain	X	p11.22	53.238	54.239	1,001	Inherited	Maternal	
14	Gain	X	q28	152.373	153.433	1,060	Inherited	Maternal	
54	Gain	X	q28	152.850	153.060	210	Inherited	Maternal	
72	Gain	X	q28	152.930	153.230	300	De novo	n.d.	

26 pathogenic imbalances detected in our series of patients with idiopathic mental retardation/developmental delay and congenital anomalies.

(a) Position of the first and last altered probes according to hg18 (UCSC)

(b) Nomenclature according to Bejerano et al. (2004). Hyphenation denotes clusters where all the consecutive UCEs are included.

When comparing gains and losses, we found an excess of deletions (10 out of 15) that contain UCEs, while only 3 out of 11 duplications bear these elements. This difference is in the limit of significance (P = 0.055; Fisher's exact text). However, when analysing separately both kinds of CNVs, it becomes rather evident that the enrichment for ultraconserved elements is mainly due to the deletions (see Table 2). Deletions show a highly significant excess of UCEs, estimated in 3.6 times more frequent than expected. Conversely, the pathogenic gains of dose that we have detected in our series do not deviate significantly in the number of ultraconserved elements contained.

**Table 2:** Analysis of frequencies of UCEs

	Size (Mb)	% of genome	No. UCEs	Enrichment ratio ^a^	P-value ^b^	No. Sno/miRNA	Enrichment ratio ^a^
Genome	3107		481			1120	
Polymorphic CNVs	23.74	0.76	0	-	0.06	7	0.82
Pathogenic CNVs	82.97	2.67	29	2.26	0.000007	42	1.41
Deletions	47.45	1.53	26	3.55	0.000001	18	1.05
Duplications	35.52	1.14	3	0.54	0.28	24	1.87

Analysis of frequencies of ultraconserved elements (UCEs) among polymorphic and pathogenic CNVs detected in our series of patients. Note that the lack of UCEs among polymorphic CNVs is in the limit of significance, while the excess of UCEs among pathogenic CNV is largely due to those present in deletions. Comparatively, similar analyses of frequencies for microRNAs/snoRNAs do not show significant differences, thus confirming the representativeness of the examined regions.

(a) Enrichment ratio represents the ratio between observed and expected elements. It is calculated as (item count/size of region)/(total items/size of genome).

(b) Chi-square test for goodness of fit.

The parental origin of the chromosome bearing the alteration could be determined in 21 cases through microsatellite segregation analyses. Ten rearrangements occurred as *de novo *events in the paternal chromosome and four in the maternal chromosome, while the remaining alterations were inherited from the carrier mother (in five cases) or from the father (two cases). These inherited CNVs were X-linked or associated to mild affectation in the carrier parent. Remarkably, 24 out of the 29 UCEs were contained in the rearrangements inherited from the father or originated in a paternally derived chromosome. However, this association might well be an indirect consequence of a higher proportion of deletions originated in the paternally derived chromosome. Both paternal and maternal deletions show a similar enrichment ratio for UCEs (3.9 and 3.0, respectively), while the paternal and maternal duplications contain a lower than expected ratio (0.9 and 0.0, respectively).

On the other hand, it is noteworthy that none element was found in our series among the 93 CNVs considered as polymorphic variants, some of them not previously reported, which altogether cover about 23.7 Mb (see Additional file 1). This absence of ultraconserved elements among benign CNVs almost reached significance (see Table 2), in accordance with previous studies described above.

## Discussion

We found a highly significant enrichment of ultraconserved elements among pathogenic imbalances causing neurodevelopmental disorders. It can be therefore suggested that these elements, or their neighbouring genes, might be considered particularly sensitive to dose imbalances.

It might be argued that the positive association between clinically relevant CNVs and UCEs merely reflects that pathogenic alterations preferentially affect gene-rich regions and, as an indirect consequence, they might contain more ultraconserved elements. In accordance, we performed a similar analysis for the gene content after differentiating between the pathogenic CNVs that contain and those that do not contain UCEs, based in the number of annotated RefSeq Genes [8], as well as in the more conservative Consensus Coding Sequence (CCDS) project [9]. As can be seen in Table 3, the UCEs-containing CNVs showed a lower than expected gene content, what allow us to reject this spurious association. Furthermore, this result is in good agreement with the previous report that non-exonic ultraconserved elements are often found in "gene deserts" [2]. It is worth mentioning that 88 of them were reported to be more than 100 kilobases away from any known gene. Conversely, those pathogenic CNVs that do not contain UCEs showed a strikingly higher than expected gene content. This result suggests that gene content and presence of UCEs might be complementary criteria of pathogenicity, that is, pathogenic CNVs tend to affect either a high number of genes or closely regulated genes near UCEs, many of them in "gene deserts". In turn, this complementarity can be interpreted as reinforcement for the association between pathogenic CNVs and ultraconserved elements.

**Table 3:** Relation between presence of UCEs and gene content

	Size (Mb)	No. Gene annotations (RefSeq)	Enrichment ratio ^a^	No. Gene annotations (CCDS)	Enrichment ratio ^a^
Genome	3107	33,244		19,841	
Pathogenic CNVs with UCEs	56.591	488	0.81	311	0.86
Pathogenic CNVs without UCEs	26.384	781	2.76	477	2.83

When comparing the observed/expected ratio of gene content in pathogenic imbalances attending to the presence or not of ultraconserved elements, it results evident that only pathogenic CNVs without UCEs show an excess of gene content. Consequently we can reject that the enrichment of UCEs in clinically relevant CNVs are due to a higher gene content.

(a) The ratio between observed and expected elements was computed as specified in table 2.

Another putative argument for a random association between pathogenic CNVs and UCEs might be derived from the fact that many UCEs appear clustered. However we found a similar number of isolated and clustered ultraconserved elements in our series (13 and 16, respectively). In fact, the isolated elements were over-represented as compared with clustered elements. In the 2.67% of the genome are present 10% of the isolated elements (13/131) and 4.6% of the clustered elements (16/350), what represent the respective enrichment ratios of 3.7 and 1.7. In any case, clustering does not modify the *a priori *probability to find these elements, because every imbalanced region represents an independent 'sampling' event. In order to check the representativeness of the CNVs detected in our series, we analysed the frequencies of other sentinel elements, microRNAs/snoRNAs, present in a similar order of magnitude in the genome than UCEs, and that also tend to cluster. These elements were evenly represented in any category of CNVs in frequencies very close to the expected (enrichment ratios near 1, see Table 2). Even the slight excess among duplications (observed/expected = 1.87) may well be interpreted as reflecting a higher content of genes.

To explain the lack of association of UCEs with duplications in our series, several factors (not mutually exclusive) may be argued, such as a patient-selection bias or the fact that duplications are on average slightly larger and clearly more gene-rich than deletions. On the other hand, there is an excess of duplications in the X chromosome. A high incidence of duplications on the X chromosome contributing to mental retardation has been recently reported [10], what might be related to the fact that the resulting gene dosage in males is higher than with any other chromosome (a dose increase of 100% instead of 50%). This special condition might also helps to explain that more genes are clinically relevant if duplicated in the X chromosome, and consequently more genes can be potentially pathogenic by duplication even when they are not tightly regulated genes, for instance by ultraconserved elements.

Although our results did not reach significance (see Table 2), we have confirmed a depletion of UCEs among benign CNVs as previously described [7]. Derty and collaborators additionally found that most of the ultraconserved elements present in benign copy number variants overlapped exons. These exonic UCEs are present in many genes encoding well-known RNA-binding proteins, while intergenic UCEs are preferentially flanked by developmental genes, particularly involved in early developmental tasks [2]. We found that both exonic and intergenic ultraconserved elements appear to be equally represented in the CNV regions associated to disease, what advocates for a dose-sensitive character of the nearby genes in either case.

In summary, we have found that pathogenic CNVs show an enrichment of ultraconserved elements, conversely to benign CNVs. It can be argued that since UCEs are often associated with genes involved in RNA processing and developmental tasks, especially these genes are dosage-sensitive and hence, a heterozygous deletion/duplication including such a gene will more likely result in disease.

## Conclusions

In the view of the association between ultraconserved elements and pathogenic dose variants, we therefore propose that these elements may be interpreted as hallmarks for dosage-sensitive genes, particularly for those genes whose gain or loss may be directly implied in neurodevelopmental disorders. Obviously, the presence of this kind of elements in CNVs of unknown consequences should be used with caution together with other pathogenicity criteria, such as the gene content or the mode of inheritance. This is an important issue given the current limitation in some instances to differentiate between pathogenic and benign rare copy number variants.

## Methods

### Laboratory analyses

Genomic DNA from 200 patients and their parents was purified by standard proteinase K/phenol-chloroform procedures. All the patients showed idiopathic mental retardation associated to congenital anomalies, non assignable to known syndromes after clinical examination by two specialists. Informed parental consent, as approved by our Hospital Review Board, was obtained prior to research studies. Patients C1 to C5 were studied by clone-based array CGH as reported elsewhere [11]. Patients M1 to M3 were detected in the initial screening for microdeletion syndromes by commercial MLPA (SALSA P245; MRC-Holland, Amsterdam), following the recommendations of the manufacturer. All the remaining cases were studied by array-based comparative genomic hybridization (human genome CGH microarray AMADID: 014950, from Agilent Technologies, Palo Alto, CA) as recommended. The patients' DNA samples were tested against a pool of 10 sex-matched normal DNA samples, all of them (patients and normal controls) from our geographical area. Confirmatory analyses and familial studies were done by microsatellite marker segregation analyses and commercial or home-made MLPA studies (primers and conditions available upon request).

The CNVs detected with both kinds of arrays were collected together, defined by the distal ends of the first and last probe altered. It is worth to note that most benign and pathogenic CNVs were detected by the commercial oligonucleotides-based array because of its higher resolution and because it was applied to 95% patients. On the other hand, they can be considered complementary, as many of the small polymorphic CNVs previously detected in the clone-based array [11] could not be refined in the oligo-array because of lack of probes in such regions, designed in order to avoid frequent polymorphic CNVs.

### Statistical analyses

Item counts for the presence of ultraconserved elements, sno/miRNAs and genes in the regions encompassed by CNVs were performed through the UCSC Genome Browser [12], based on build hg18. The delimiting positions of all the ultraconserved elements, tracked in the hg16 version, were converted to built hg18 through the 'Convert' Feature http://genome.ucsc.edu/goldenPath/help/hgTracksHelp.html#Convert and compiled in an in-home excel sheet to facilitate visual inspection. In every case, a confirmation of the positions was performed by employing as reference the previously known delimiting genes in the CNVs. Items contained in overlapping rearrangements were considered once, avoiding duplications of items or sizes.

In order to measure the strength of association, we employed the observed/expected ratio (called 'enrichment ratio' in tables 2 and 3). The expected frequencies were computed as the product of the total number of elements (for instance, n = 481 UCEs) by the proportion of genome examined (p_i _= ∑_i_Mb/Mb_genome_). Frequency analyses were performed by the Pearson's chi-square goodness-of-fit test, employing the observed and expected frequencies previously computed.

## Competing interests

The authors declare that they have no competing interests.

## Authors' contributions

FM conceived the study, participated in its design and coordination and drafted the manuscript. SM participated in the molecular genetic studies and helped in the data collection. MR participated in the design of the study, in the data collection and performed the statistical analysis. SO participated in the molecular genetic studies. DB participated in the molecular genetic studies and in the draft of the manuscript. RQ participated in the molecular genetic studies. SM participated in the design of the study and helped to perform statistical analysis. CO participated in its conception, design and coordination and helped to draft the manuscript. All authors read and approved the final manuscript.

## Pre-publication history

The pre-publication history for this paper can be accessed here:

http://www.biomedcentral.com/1755-8794/3/54/prepub

## Supplementary Material

Additional file 1 **Polymorphic imbalances**. Polymorphic imbalances detected in our series of patients with idiopathic mental retardation/developmental delay and congenital anomalies. There are a total of 93 different CNVs present in 115 cases. These CNVs have previously been reported as polymorphisms and/or are present in at least one healthy member of the family. Altogether, these CNVs span a total of 23.74 Mb, excluding the overlapping regions. Note that all these regions do not contain any ultraconserved element. Click here for file
